# The Structure and Composition of Puerto Rico’s Urban Mangroves

**DOI:** 10.3390/f11101119

**Published:** 2020-10-21

**Authors:** Benjamin L Branoff, Sebastián Martinuzzi

**Affiliations:** 1 Gulf Ecosystem Measurement and Modeling Division, Office of Research and Development, United States Environmental Protection Agency, Gulf Breeze, FL 32561, USA; 2 SILVIS Lab, Department of Forest and Wildlife Ecology, University of Wisconsin-Madison, 1630 Linden Drive, Madison, WI 53706, USA

**Keywords:** novel forests, urban disturbances, anthropocene, temperature stress, eutrophication

## Abstract

This study characterizes the structure and composition of mangrove forests across urban gradients in Puerto Rico. It then uses a suite of hydrologic, water chemistry, and land cover variables to test for the relative importance of urban intensity alongside flooding and water chemistry in explaining observed variability in forest structure and composition. Three separate statistical tests suggest a significant but limited influence of urbanness on forest composition and structure. In the most urban sites, the diameters of the largest trees were 27% larger, but all structural measurements were best explained by surface water chemistry, primarily nitrogen concentrations. Concentrations of ammonium and total Kjeldahl nitrogen best explained stem density, tree girth and canopy height. The most urban forests also contained 5.0 more species per hectare, on average, than the least urban forests, and simple regression suggests that urban metrics were the most powerful predictors of forest composition. The most urban forests were more dominated by *Laguncularia racemosa*, while both *Avicennia germinans* and *Rhizophora mangle* were found to be less abundant in the most urban sites, a trend that may be linked to the influence of precipitation and tidal connectivity on porewater salinity across the urban gradient. In multiple regression, no statistical difference was detected in the importance of surrounding land cover, flooding, or water quality in explaining the variance in either composition or structural metrics. This suggests that while a given forest metric may be strongly linked to either land cover, water quality, or flooding, all three are likely important and should be considered when characterizing these forests. With more human dependents in urban areas, the provisioning of important ecosystem services may be influenced by land use variables in addition to the more commonly measured metrics of water chemistry and flooding.

## 1. Introduction

Urbanization has been an important contributor to forest disturbance at the turn of the twentieth century, with trends indicating an increasingly important role in tropical coastal areas over the next few decades [1–4]. The conversion of forests to developed lands usually follows economic transitions that favor industrialized societies and continuing transitions may in some cases support regrowth, albeit with novel forests [5, 6]. As a result, forests in urban and surrounding lands are often represented by unique anthropogenic influences and ecological traits [7–9].

Characterizations of urban forests often consist of lower stem densities and larger individuals, with stands exhibiting increased edge openness and regeneration failure [8]. These forests are further generalized as having higher floral diversity, usually due to non-native species from residential gardens and municipal landscaping. As a result, the function of these systems is also novel, as evidenced by altered community composition, biogeochemistry, productivity, and resiliency [10, 11]. This, in turn, gives way to an adjusted provisioning and valuation of ecosystem services [12, 13]. Much of this is known from the study of terrestrial systems, with comparatively little understood of mangroves, the dominant forested biome of tropical, sub-tropical, and warm temperate estuaries.

Globally, mangrove coverage in the largest cities is decreasing faster than overall rates from corresponding countries [14]. In some cases, this loss results in fragmented forests consisting of novel species assemblages and size classes [15–18]. There are cases of expanding urban mangrove coverage [19, 20], although the young forests may contain less biomass [21]. These studies suggest a systemic influence of urban land-use on mangrove structure and composition, but most were done independently of hydrology or water chemistry, which have long been recognized as powerful influences on the same structural and compositional metrics [21–23].

Flooding metrics of hydroperiod and flood frequency are important for mangrove seedling survival and adult growth [24, 25]. Water salinity, pH, dissolved oxygen, phosphorus, and nitrogen concentrations are also known to influence mangrove physiology and forest structure and composition [26–30]. While there are some anecdotal connections between mangrove hydrology, water chemistry, and urbanness [31–33], quantified connections between these components are absent or weak, leaving little definitive evidence for the hypothesis that urban land use influences mangrove forests through hydrology and water chemistry.

In Puerto Rico, Brandeis, et al. [34] provide a limited structural inventory of the mangrove and non-mangrove forests of the island’s largest city, San Juan, based on a small sample area, but there are no conclusions regarding the influence of urban areas on this structure, and much less is known of the urban mangroves outside of the San Juan metropolitan area. Overall, urban mangrove coverage in Puerto Rico has largely failed to expand during the last quarter of the twentieth century, despite a 12% increase in mangroves across the island [35]. Furthermore, urban forests were found to have fewer and smaller forest fragments compared to more rural sites. However, while there is some limited information on the structure and composition of these forests, there is little empirical evidence tying any observed changes in mangrove ecology to specific components of urban landscapes. Furthermore, as the 2017 hurricane season significantly disturbed these forests [36], it is important to have a pre-disturbance benchmark by which to monitor and compare subsequent recovery among the urban and non-urban forests.

In addition to the above shortcoming, there are no universally accepted definitions for “city” or “urban”, and thus little consensus on what defines an urban forest and our understanding of their structure and function [37, 38]. Many of the above described studies use qualitative comparisons of urban vs. non-urban but provide no definitions for these characterizations, and a very limited few attempts to quantitatively define urbanness through landcover (e.g., impervious surfaces) or demographics (e.g., population density). As a result, it is difficult to repeat or synthesize studies to understand systemic patterns in urban forest ecology. Thus, urban ecologists increasingly recognize the need for quantitative metrics of urbanness that can be represented as an urban gradient [11, 39, 40]. Such metrics not only allow for a more causal understanding of urban ecosystem patterns towards hypothesis testing, but also for more repeatable studies across multiple urban landscapes.

This study uses a combination of ground-based and remote sensing measurements to address the following objectives: 1) Characterize the structure and composition of mangrove forests along quantified urban gradients in three watersheds of Puerto Rico, 2) Test the hypothesis that urban forests are characterized by lower stem densities and stand biomass, larger individuals, and higher floral diversity when compared to less-urban counterparts, and 3) Test for the combined importance of urbanness, hydrology, and water chemistry on mangrove forest structure and composition through multiple regression. Addressing these objectives will be an important component of mangrove management under increasing pressure from urbanization in the Caribbean and worldwide [4, 41–43].

## 2. Materials and Methods

### 2.1. Study Location

**Figure 1.**
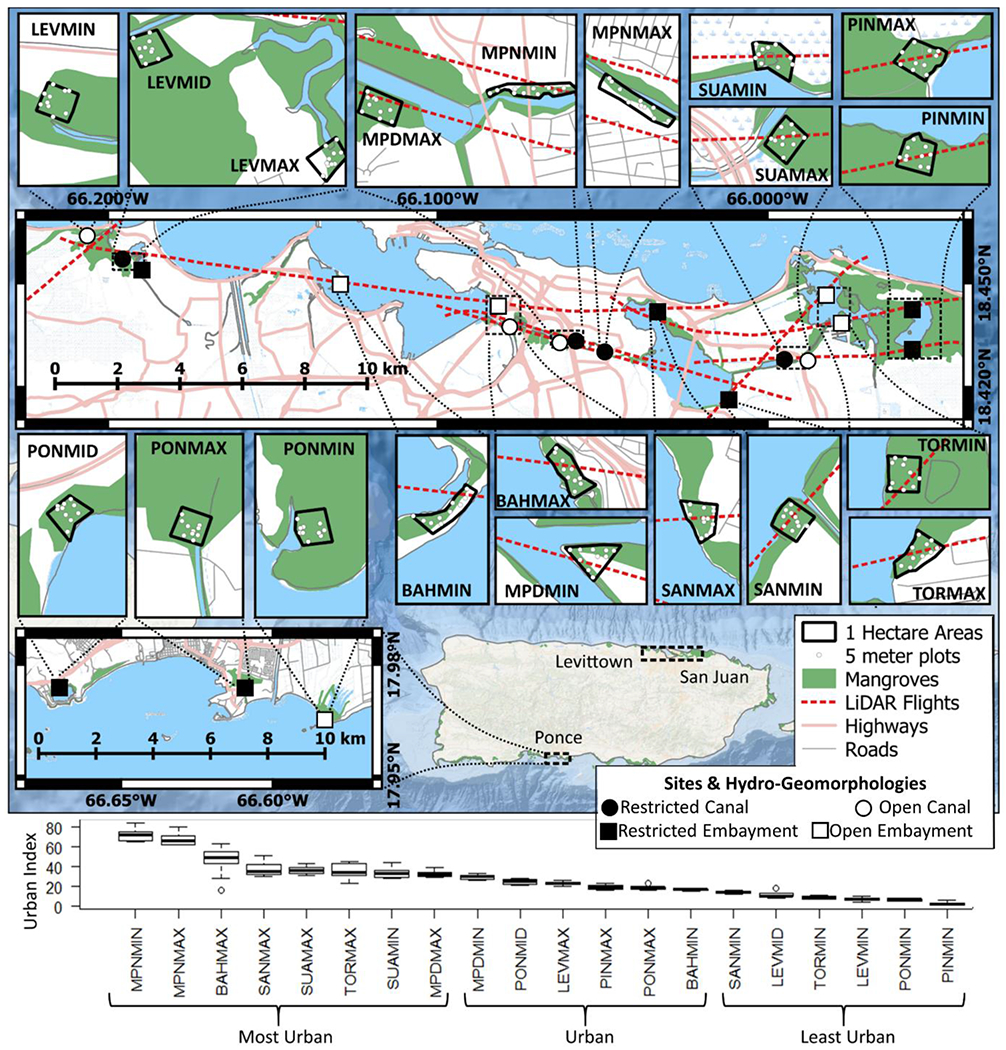
Study sites are twenty 1-hectare mangrove forests in three watersheds (top) and spanning a gradient of urbanization as measured by the urban index (bottom). The urban index is a combination of urban land use, vegetation and open water coverage, population density, and road length. A value of 0 represents the least urban site and a value of 100 is the most urban site. “MAX” and “MIN” postscripts refer to urbanization levels within each water body. BAH is the San Juan Bay, MPN is the Caño Martín Peña, SAN is the San José lagoon, SUA is the Suarez canal, TOR is the Torrecilla lagoon, PIN is the Piñones lagoon, LEV is Levittown and PON is Ponce.

We focused on twenty mangrove sites (one-hectare each) from Branoff [45] and distributed in the coastal areas of Puerto Rico. These sites were established according to a previously developed urban index [44], such that they fell within the greatest range of urbanness (Figure 1, Table 1). The urban index is a unitless metric of urbanness that incorporates information on urban land use, vegetation and open water coverage, population density, and road length, where a value of 0 represents the least urban site and a value of 100 is the most urban site (Table 1). Fourteen sites fall within the San Juan Bay Estuary and three sites each fall within watersheds in Ponce and Levittown (Figure 1). Sites in the San Juan Bay Estuary are named for their waterbodies (BAH is the San Juan Bay, MPN and MPD are the un-dredged and dredged portions, respectively, of the Caño Martín Peña, SAN is the San José lagoon, SUA is the Suárez canal, TOR is the Torrecilla lagoon, and PIN is the Piñones lagoon). In Ponce and Levittown, mangrove area is comparatively smaller and so sites are named for their watershed only: LEV is Levittown and PON is Ponce. To ensure we sampled the greatest feasible range of urbanness in a given area, two sites per waterbody in the San Juan Bay Estuary and three sites each in Ponce and Levittown were selected, each representing the relative minimum, mid, or maximum urban index values for that area. In the San Juan Bay Estuary, two sites were located in the minimum and maximum levels of urbanness in each waterbody, as denoted by “MIN” and “MAX” following waterbody abbreviations in site names. In Levittown and Ponce, three sites were placed at the minimum, median, and maximum urbanness levels of each watershed, as denoted by “MIN”, “MID” and “MAX” following watershed (“PON” and “LEV”) abbreviations. Thus, “MIN”, “MID” and “MAX” in site names are relative to local urbanness levels only, not island wide. All sites are bound on one side by a waterbody and most sites are roughly 100 m by 100 m, with some asymmetry due to non-linear coastlines. In some cases (BAHMIN, BAHMAX, MPDMIN, MPNMIN, MPNMAX, TORMAX) forests were constricted by natural or anthropogenic features and these sites were extended along the shoreline to compensate for a lack of forest less than 100 m from the shore.

Human use of the mangroves is minimal at most sites, although harvesting of the crustacean, *Cardisoma guanhumi*, is sparsely evident at some and forest use for grazing and corralling of horses, pigs, and dogs is also apparent at MPNMAX and LEVMAX. There was no evidence of wood harvesting at any of the sites. For more information on the urban index and plot information see Branoff [44].

The mangroves of San Juan were most recently described by Brandeis, Escobedo, Staudhammer, Nowak and Zipperer [34] as being dominated by *Rhizophora mangle*, although the authors point out this is likely due to sampling methods and that *Laguncularia racemosa* best characterize the forests and represents more biomass than any other species. Very little has been published on the mangroves of Levittown, which surround an estuary composed primarily of an artificial tidal lagoon constructed to drain surrounding settlements and connected to the ocean through a tidal creek and permanent inlet. Unlike the other two sites, the mangroves at Ponce are largely unconnected and do not share the same estuarine conditions among them. Additionally, Ponce receives a much drier southern climate in comparison to the northern sites, with a median annual rainfall of 755 mm versus 1600 mm on the northeastern coast [44]. Like Levittown, very little is known about the mangroves in Ponce, although one study did characterize the hydrology of Punta Cabullones [45].

All sites were classified as one of four hydro-geomorphologies following aerial imagery, previous studies, and the results of water level monitoring from Branoff [44]. These classifications are embayments (i.e., ocean, bay, or lagoon) or canals, and either open or partially restricted to tidal exchange. Due to a lack of tidal connectivity, water levels in partially restricted sites respond more strongly to rainfall events in comparison to open sites [44] and this is the primary criteria for their hydro-geomorphic classifications although the following diurnal water level ranges also reflect respective tidal connectivities. Ocean and bay sites always have direct tidal exchange and include the two San Juan Bay sites BAHMIN and BAHMAX, and the ocean site in Ponce, PONMIN. Hydrographs of these sites show mean diurnal ranges of 40, 47, and 14 cm, respectively [44], reflecting that of offshore buoys in the Atlantic Ocean (48 cm) and Caribbean Sea (20 cm) [46, 47]. Lagoon sites, however, may include embayments that are partially restricted to tidal flow. The only open lagoon sites are in Torrecilla lagoon (TORMAX and TORMIN), due to its dredged mouth at Boca de Cangrejos (Ellis 1976), as evident in a mean diurnal range of 41 cm [44]. Closed lagoons in the San Juan Bay Estuary include Piñones (PINMAX and PINMIN) and San José (SANMIN and SANMAX), whose mean diurnal ranges are reduced to 3 and 4 cm, respectively [44]. Elsewhere, closed lagoons include the Levittown lakes (LEVMAX) in Levittown, and in Ponce the Salinas lagoon (PONMID) and the Port of the Americas (PONMAX), with mean diurnal ranges of 0, 5, and 3 cm, respectively [44]. Canal sites may also have open or partial tidal exchange. Open canals are the east end of Suárez canal, SUAMAX, which shares the dredged connection with Torrecilla and has a mean diurnal range of 17 cm, the west and dredged portion of the Caño Martin Peña, MPDMIN and MPDMAX, both with mean diurnal ranges of 47 cm, and the mouth of the Río Cocal in Levittown, LEVMIN, with a mean diurnal range of 13 cm [44]. Partially restricted canals are that connecting the Río Cocal to the Levittown lakes, LEVMID, with a mean diurnal range of 12 cm, the undredged eastern portion of the Caño Martín Peña, MPNMAX and MPNMIN, each with diurnal ranges of 27 cm and the portion of the Suárez canal restricted by the Baldorioty expressway (SUAMIN), which shares the San José range of 4 cm [44].

### 2.2. Ground-Based Measurements

Ten subplots within each one-hectare site were sampled, each being a 5 m radius circle of 78.5 m^2^. A 5 m radius plot design was used as recommended for small stem forests [48]. Plots were established by fixing the end of a 5 m rope at ten randomly generated coordinates within each site and extending the rope until tight. These center points were located using a Garmin eTrex 10 global positioning system with an accuracy of ± 3 m. All woody plants greater than 1 cm in diameter at the breast and within the 5 m radius were identified and their diameter measured with a diameter measuring tape at a height of 1.4 m, or diameter at breast height (dbh). For individuals of *R. mangle* whose primary prop roots joined the trunk at heights greater than 1.4 m, trunk diameter was measured just above the confluence of the prop roots where a true mainstem existed. Branches from mainstems of all species were also measured if they originated at heights less than 1.4 m and if their dbh also exceeded 1 cm. Finally, in addition to the surface water chemistry metrics described below, we also took porewater salinity measurements at each site by extracting a 5 mL sample of porewater-sediment mix through the use of a small diameter tube connected to a 50 mL syringe similar to that described by McKee, et al. [49]. This was performed at depths of 0, 10 and 20 cm, and the resulting extraction was allowed to settle until a clear sample of water could be measured for salinity, in ppt, via a VEE GEE STX-3 refractometer. In some cases, a clear sample of water could not be obtained from the sediment and no salinity was recorder for that depth at that site.

Aboveground biomass estimations were calculated for each tree through allometric equations using the measured dbh and wood specific gravities when available. Halophytic plant types and salinity tolerances, when available, were provided by Santos, et al. [50]. For the three true mangrove species (*A. germinans*, *L. racemosa* and *R. mangle*), equations were species and dbh specific as derived from three separate sources on Caribbean mangroves [51–53]. The mean of these three values was used and when no value was available for greater size classes, a general equation for mangrove habitats was used from Chave, et al. [54]. This equation was also used for non-mangrove species in combination with specific gravities derived from Reyes, et al. [55]. All ground measurements used to characterize mangrove structure and function are described in Table 2.

### 2.3. LiDAR

Airborne LiDAR data were collected over parts of Puerto Rico in March of 2017 using the NASA G-LiHT (Goddard’s LiDAR, Hyperspectral and Thermal) imaging system [56], as part of the US Department of Energy, Next-Generation Ecosystem Experiments-Tropics project (https://ngee-tropics.lbl.gov) (Figure 1). LiDAR data provide detailed information on forest 3D structure and therefore can be used to quantify forest structure across urban gradients [57, 58]. Among our sites, LiDAR data from the GLiHT campaign were only available for the San Juan Bay Estuary sites and the LEVMID site. Data were collected at a flying altitude of 335 m above ground and ~12 pulses m^−2^. Point clouds were processed in R version 3.6.1 [59] and the lidR [60] and rLiDAR [61] packages. The LiDAR point clouds were clipped to the 5 m radius plot boundaries using the *lasclip* function and height and canopy metrics extracted from the *LASmetrics* function. Common LiDAR metrics returned by *LASmetrics* were derived for each plot, including: mean height, standard deviation of height, height percentiles, skeweness, canopy cover, and canopy density (see Table 2 for more information). We used the 90th height percentile to represent the maximum canopy height throughout the analysis to avoid outliers associated with actual maximum return heights [62]. The geolocation error of the field plots (± 3 m) should not be a problem, as the LiDAR data was used to characterize the sites, and not to relate the LiDAR data and field data at the plot level.

### 2.4. Land Use, Flooding, and Water Chemistry

To explain the variability in the above described forest structure and composition metrics, we used several land use, flooding, and water chemistry variables as outlined in Table 3. Because analysis at multiple scales is important and ecological effects of land cover are typically most pronounced within 1 km [63, 64], land cover data were cropped, masked, and sampled using circles of radius 50-1000 m around each plot. For each circle we calculated the extent of three classes: urban (including the classes Impervious and Developed Open Space, respectively), green and blue area (represented by the sum of all vegetation and open water classes), and mangrove, corresponding to estuarine scrub/shrub wetland and estuarine forested wetlands. This was accomplished through the *st_buffer*, *st_crop*, and *st_mask* functions of the sf package [65] and the *getValues* function from raster [66].

Road length was calculated by clipping a roads layer and summing the length of all resulting road segments. Population density was calculated from the 2010 Census and the land cover and road layers previously described by assuming people lived only in non-road impervious surfaces. These variables describing land cover, population, and roads are included in a single urban index that represents the relative urbanness of each site [44]. The urban index first standardizes each variable from 0 to 100, 0 being the least urban and 100 being the most urban value, then computes an average of each normalized variable to give the single urban index representing a combination of all variables [45]. In addition, we used information on site hydrology and water quality derived from water level models and surface water quality measurements representing the period from 2012 to 2017 at the same study sites [44]. Water level models were constructed from water level observations recorded by data loggers at each site and precipitation observations at nearby weather stations. Tidal harmonics models were used to extract tidal constituents and moving sums were used to model precipitation contributions to observed water levels. These were then used to reconstruct water levels over a five-year period in which flooding conditions were assessed every hour by comparing the predicted water level to the elevation of the forest as determined from digital elevation models [45]. Hydrology metrics include average depth (m), proportion of time flooded (fraction), mean daily flood frequency (floods day^−1^), mean flood length (days), number of days with at least one flood per year (days), flooded hours per year (hours) and dry hours per year (dry). Water chemistry measurements were made in surface waters within 1 km of each site on a monthly and bi-annual basis over the same five-year period by the San Juan Bay Estuary Program and are available at https://estuario.org/. These measurements were only available for the fourteen sites in San Juan. Monthly measurements are obtained in-situ by a handheld sonde and are dissolved oxygen (mg/L), pH, salinity (PSS), specific conductivity (ms/cm), and temperature (°C). Bi-annual measurements are obtained by water samples from surface waters and laboratory chemical analyses. These include ammonium (mg/L), total Kjeldahl nitrogen (mg/L), nitrate and nitrite (mg/L), and phosphorus (mg/L). A detailed description of the water quality and hydrology measurements is given in Branoff [44] and scripts and results for this analysis can be found at https://github.com/BBranoff/Urban-Mangrove-Hydrology.

### 2.5. Analyses and Statistical Testing

All numerical analyses and statistical tests were performed in R version 3.6.1 [59]. Except for regression analyses, statistical tests were performed at the site and watershed levels using values from each plot (*n* = 200; 10 plots for each of the 20 sites). Comparisons across sites and watersheds were done through an ANOVA as calculated by the *aov* function of the stats package [59], and pairwise tests were performed through a Tukey Honest Significant Differences test as computed through the *TukeyHSD* function in the same package. Non-metric multidimensional scaling (NMDS) was performed to observe the relative differences in forest structure and composition between sites and hydro-geomorphologies (i.e., open embayment, restricted embayment, open canal, restricted canal), and to observe the potential contribution of the urban index to any separation and/or grouping of forests. This was performed twice, once on compositional measurements (e.g., species diversity, percent of biomass as *R. mangle*, etc.) and again on structural measurements (e.g., maximum dbh, stem density, biomass etc.). Functions for this analysis are from the vegan package [70]. NMDS was first performed through the *metaMDS* function with the default of two dimensions. Results were then rotated through the *MDSrotate* function so that the horizontal axis of the NMDS was aligned with the vector describing the urban index. Vectors describing the various measurements were then calculated through the *envfit* function, which finds the projection of the maximum correlation between each variable and the ordination. Finally, ellipses describing the 95% standard error confidence area for each hydro-geomorphology were drawn using the *ordiellipse* function. Sites in the NMDS were grouped into hydro-geomorphic characterizations as reported in Branoff [44] to account for any potential groupings due to differences in flooding dynamics.

The contribution of environmental predictor variables (e.g., urban cover, proportion of time flooded, ammonium concentration, etc.) on forest structure and composition (e.g., stem density, biomass, number of species, etc.) was analyzed through both simple and multiple regression. To single out the most important environmental influences on forest metrics, simple regression models were constructed through the *lm* function [59] in the form y~x, y~ln(x), and y~ln(x+1) when environmental predictor variables included values less than or equal to 0. The highest performing models were selected as those with the highest R^2^ value whose p-value was lower than 0.05. Regression was done using mean site values to better highlight general trends across sites. These are accompanied by boxplots comparing the same forest composition and structure metrics between the least urban, urban, and most urban plots.

To examine the combined and relative importance of urban, hydrology, and water chemistry variables in explaining variability in forest structure and composition, multiple regression models were constructed by including one variable from each of the three potential influences of land cover, hydrology, and water chemistry. These models took the form of

$$\text{Y}=\text{a}+{\text{b}}_{1\times 1}+{\text{b}}_{2}{\text{X}}_{2}+{\text{b}}_{3}{\text{X}}_{3}$$

where Y is a given response variable of forest structure and composition, a is the intercept of the model, b are the predictor specific slopes, X are the predictors, and the subscripts 1, 2, and 3 represent groups of predictors from land cover, hydrology, and water chemistry. Models were constructed in the same way as simple regression, with both raw predictor variables as well as their natural logarithm to test for both linear and non-linear relationships. Bayesian Information Criteria (BIC) were calculated for all candidate models of the same response variable through the *glmulti* function of the same package name [71]. BIC was used because it imposes a higher penalty than AIC for models with multiple predictors, thus selecting for the simplest model. Collinearity in regression predictors was tested for through the *ols_vif_tol* function of the olsrr package [72], with any variance inflation factor (VIF) values greater than 4 prompting closer inspection of the model. The top performing significant model (*p* < 0.05) for each response variable were selected based on the lowest BIC value. The relative importance of each variable to final models, as determined by its contribution to the overall R^2^, was calculated through the *calc.relimp* function of the relaimpo package [73].

Graphs were produced through ggplot and the *geom_violin*, *geom_bar*, and *geom_point* functions for violin, bar and scatter plots, respectively [74]. Violin plots represent data at the plot level. Scatter plots represent data at the site level, and simple regression models were plotted through the *stat_smooth* function using the formula y~x, unless it was outperformed by the formula y~ln(x) or y~ln(x+1) as determined by the highest R^2^ value. R scripts as well as all required raw data can be found on the Open Science Framework project page: https://osf.io/uk2fd/. This includes individual tree measurements and plot level characterization of land cover, water quality, and flooding metrics, as well as LiDAR metrics at the plot level. The 2017 LiDAR data for Puerto Rico are available at https://gliht.gsfc.nasa.gov/.

## 3. Results

### 3.1. Structure and Composition of Mangroves

Nine-thousand three-hundred and eighty-one stems belonging to 7250 mainstems were identified to a total of 30 different species, resulting in an overall stem density of 5997 ± 291 stems per hectare (Table 4). Species include all three true mangrove species of Puerto Rico in addition to five other hydrohalophytic non-true mangrove species, three psammophilic species, and an additional nineteen species with undescribed halophytic characteristics. Fifty-one percent of stems were represented by *Laguncularia racemosa*, 29% were *Rhizophora mangle*, 9% were *Avicennia germinans*, 7.5% were *Thespesia populnea*, 1% was *Calophyllum spp.*, and the remaining 2.5% were made up of 25 other species. Basal area was 37.6 ± 1.4 m^2^/ha on average, 63% of which was represented by *L. racemosa*, 21% by *R. mangle*, 7% by *A. germinans* and 9% by other species. The forests held 206 ± 8.9 Mg/ha of aboveground woody biomass on average, with similar percentages by species as those of basal area.

**Figure 2.**
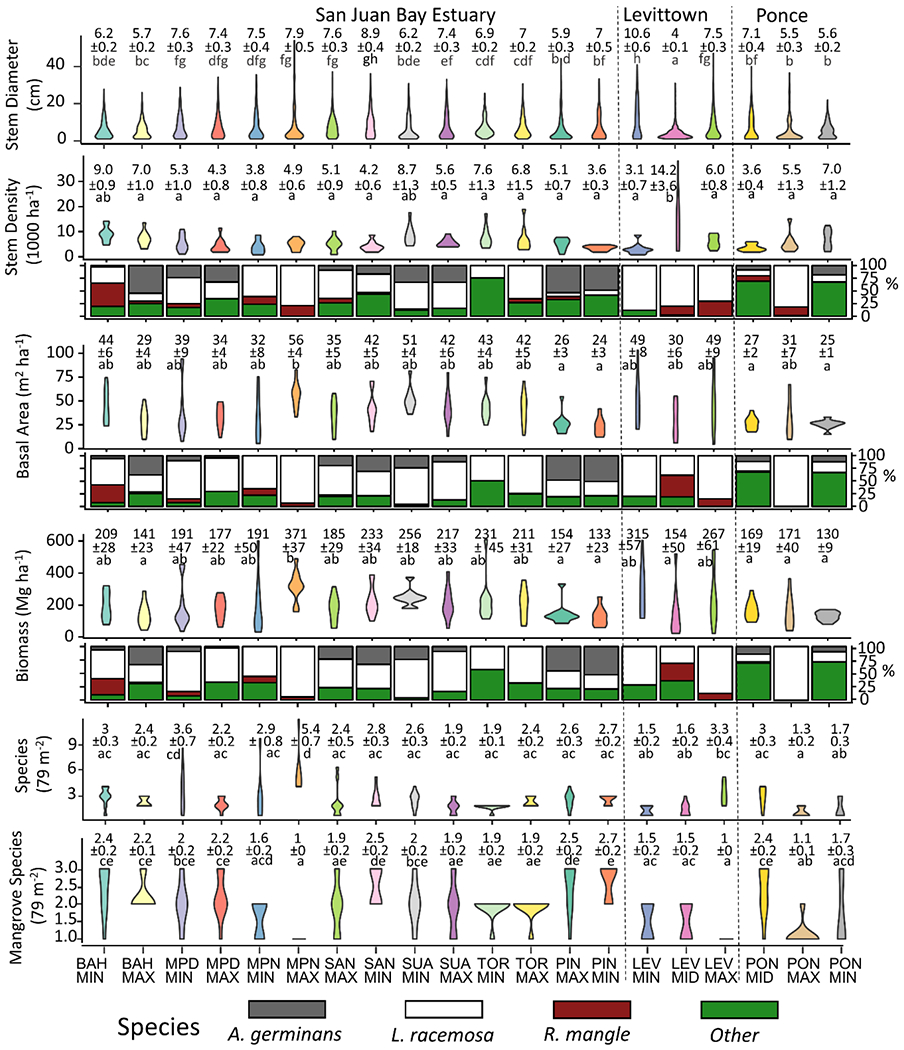
Forest structure and composition based on ground measurements. Violins are colored by site, while bar plot colors indicate species and show the relative proportion of each to the above violin plot. Most sites are dominated by *L. racemosa*, which was the only mangrove species in some of the most urban sites. Annotations are means and standard errors, and different letters denote statistical differences.

**Figure 3.**
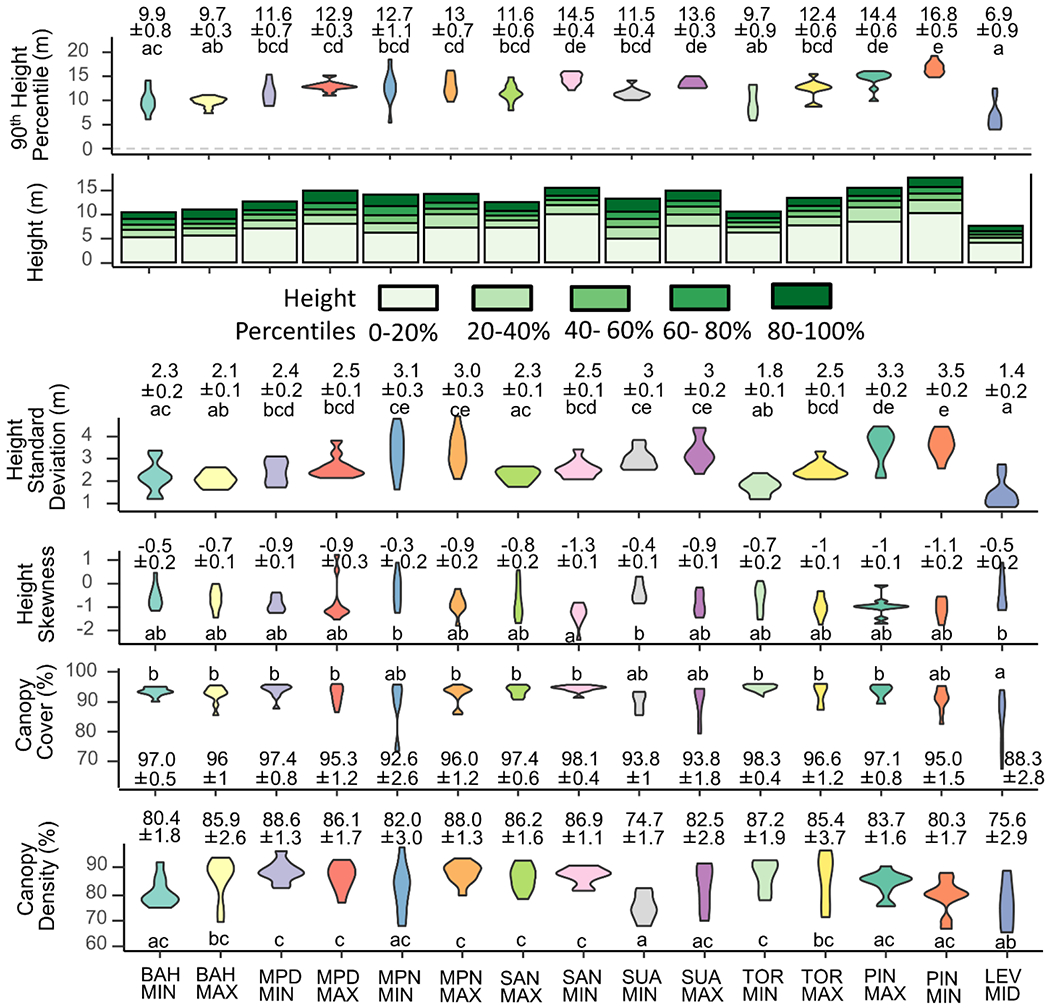
Forest structure based on LiDAR measurements. Violin plots show the distribution density of each variable, with wider portions representing higher density. Annotations are means and standard errors, and different letters denote statistical differences. Violins are colored by site, while bar plots of heights are colored to show the distribution of height by percentiles.

There were significant differences in dbh, stem density, basal area and biomass among the three watersheds. The mean dbh at Levittown was 1.9 cm smaller than that at Ponce (ANOVA; *p* = 0.05), while the stem density at Levittown was 2368 and 1966 more per hectare than that at Ponce and the San Juan Bay Estuary, respectively (ANOVA; *p* = 0.06 and *p* = 0.04, respectively). Basal area at Ponce was 14.8 and 11.0 m^2^/ha less than at Levittown and the San Juan Bay Estuary, respectively (ANOVA; *p* < 0.01 and *p* = 0.01, respectively). In biomass, Ponce held 89.5 Mg/ha less than that at Levittown (ANOVA; *p* = 0.02). At the site level, LEVMIN contained trees with a mean dbh on average 5.8 cm greater than trees at all other sites except MPDMAX, MPNMIN, MPNMAX, SANMIN, SANMAX, and TORMIN (ANOVA; *p* < 0.05) (Figure 2). LEVMID harbored 8955 more stems per hectare on average than all other sites except BAHMIN and SUAMIN (ANOVA; *p* < 0.05). In basal area and biomass, only MPNMAX differed significantly and held on average 30.8 m^2^/ha more basal area than PINMAX, PINMIN, PONMID, and PONMIN, and 217.7 Mg/ha more biomass on average than BAHMAX, MPDMAX, PINMAX, PINMIN, LEVMID, PONMID, PONMAX, and PONMIN (ANOVA; *p* <0.05). Three sites in the Caño Martín Peña, MPNMAX, MPNMIN and MPDMIN held the most species at 16, 11, and 11, respectively, while TORMIN and LEVMIN each held only two species per ha. At the plot level, MPNMAX contained an average of three more species per 78 m^2^ plot than all other sites except MPDMIN, which in turn held on average of two more species than LEVMIN, LEVMID, PONMAX and PONMIN (ANOVA; *p* < 0.05). LEVMAX and MPNMAX, both highly urban sites, contained the least number of mangrove species (one) and were statistically less diverse than half of the other sites (ANOVA; *p* < 0.05). PINMIN, the least urban site, was distinct in harboring high mangrove diversity at each plot, with an average of 2.7 species and was significantly more diverse than seven (35%) of the other sites (ANOVA; *p* < 0.05). LiDAR data were only available for sites in the San Juan Bay Estuary and for LEVMID (Figure 3). At the plot level, the mean 90th height percentile was 12.0 m, ranging from 4.0 to 19.3 m. The 90th height percentile at LEVMID was 5.5 m lower on average than all other sites except BAHMAX, BAHMIN and TORMIN (ANOVA; *p* < 0.01) while that at PINMIN was 5.1 m higher on average than all sites except SANMIN, SUAMAX and PINMAX (ANOVA; *p* < 0.05). Similarly, the standard deviation of heights was on average 1.4 m lower at LEVMID than all other sites except BAHMIN, BAHMAX, SANMAX and TORMIN (*p* < 0.05), while that at PINMIN was 1.4 m greater than all other sites except MPNMIN, MPNMAX, SUAMAX, SUAMIN, and PINMAX (ANOVA; *p* < 0.05). Mean canopy cover was 95%, with a minimum of 71% and a maximum of 99.8%, and the same values for canopy density were 83.3%, 65% and 98%, respectively. Canopy cover was 8.4% lower on average at LEVMID than all other sites except BAHMAX, MPNMIN, SUAMIN, and SUAMAX (*p* < 0.05). LEVMID was also characterized by canopy density values that were on average 11.3% lower than half of the other sites, and SUAMIN values were, on average, 12.1% lower than half of the other sites.

### 3.2. Non-Metric Multidimensional Scaling

**Figure 4.**
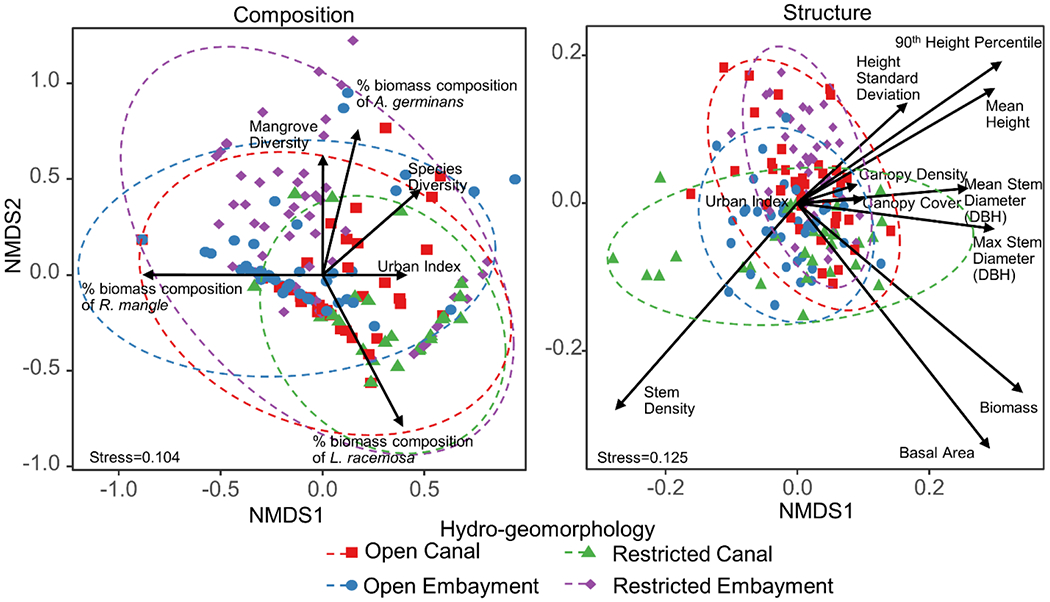
Non-metric multidimensional scaling of sites based on composition (left) and structure (right) measurements. Symbols are individual plots represented by their corresponding hydro-geomorphologies and ellipses are 95% standard error confidence ellipses for each group. Arrows are the directions of positive change for each structural and compositional measurement, as well as for the urban index, with the length proportional to the R^2^ of each vector’s fit in the ordination. There is little grouping of sites, suggesting sites are relatively similar based on the provided metrics. The urban index has the lowest R^2^ value of all variables, suggesting it contributed relatively little to the variation in forest composition and structure. Still, vector directions indicate the most urban forests are associated with higher diversity, higher dominance of *L. racemosa*, and larger and taller trees. Stress values of 0.1 on the two-dimensional analysis indicate fair to weak representations of the actual distances between sites for both compositional and structural metrics.

The NMDS showed little separation of sites or hydro geomorphologies based on the compositional and structural metrics, suggesting all sites are relatively similar in composition and structure (Figure 4). Stress values in NMDS scaling were 0.104 for compositional measurements and 0.13 for structural measurements, suggesting fair representations of actual data [75]. Although all variables were statistically significant when regressed on the ordination axes, the coefficient of determination (*R*^2^) of these fits were lowest for the urban index (*R*^2^ = 0.03 for composition and 0.05 for structure). Species diversity, percent stand biomass composition of *L. racemosa*, height, dbh, and canopy cover metrics were most aligned with the urban index, suggesting a correlation between these variables.

### 3.3. Simple Regression

**Figure 5.**
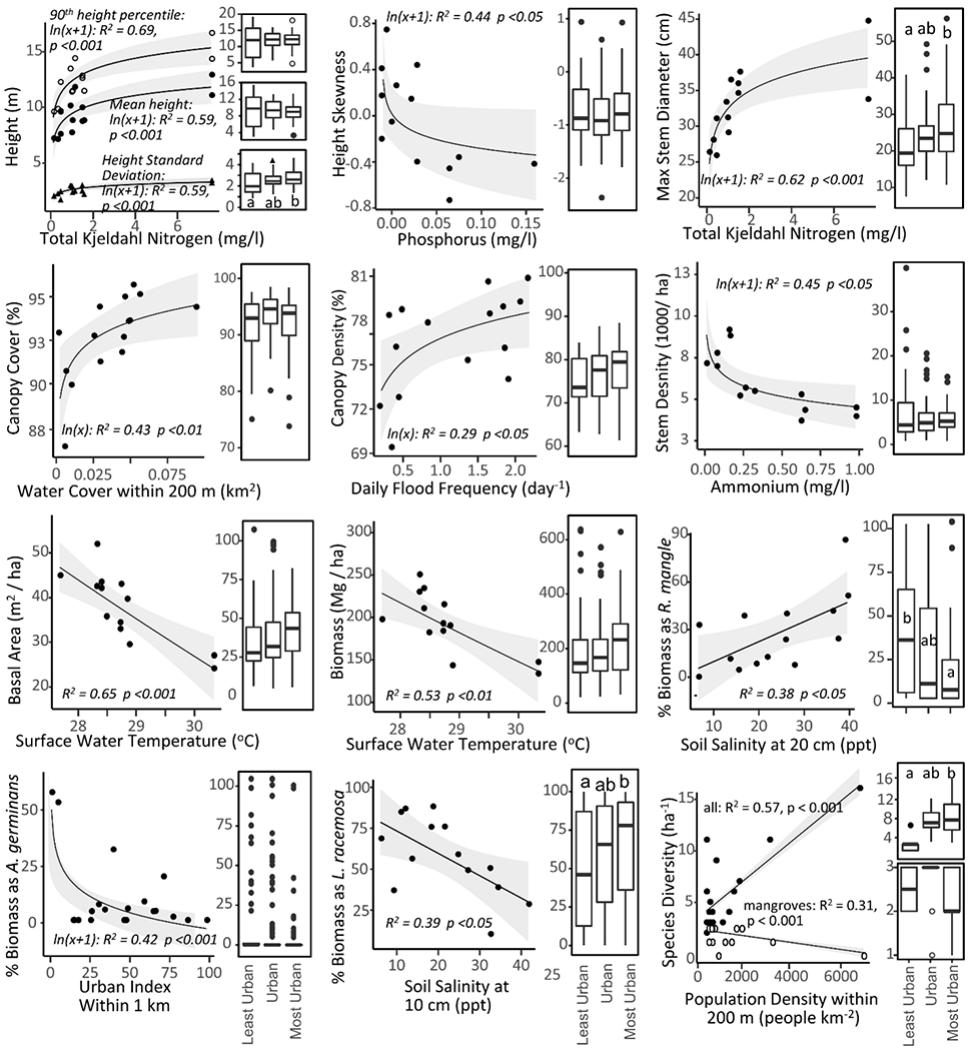
Mean site forest structure and composition measurements with their strongest predictor variables from the analysis, accompanied by boxplots of least urban, urban, and most urban plots. Letters above boxes denote statistical differences. In tree height as a response to total Kjeldahl Nitrogen (top left), open circles, filled circles, and triangles correspond to: 90th height percentile, mean height and SD of height, respectively. In species density as a response to population density (bottom right), open circles and filled circles denote overall tree species and true mangrove species, respectively. Flooding dynamics, surface water nitrogen concentrations, porewater salinity, and surrounding land cover are all strong predictors of individual forest structure and composition metrics. Shaded areas are 95% confidence intervals.

In simple regression, forest composition was best described by models including the urban index, population density, and porewater salinity, whereas structural metrics were best explained by models involving flooding and surface water chemistry predictors (Figure 5). The percent stand biomass composition of *A. germinans* decreased with increasing urban index, while that of *L. racemosa* and *R. mangle* decreased and increased, respectively, with increasing porewater salinity. Furthermore, while the percent stand biomass composition of *L. racemosa* was significantly greater in the most urban sites than the least urban sites, that of *R. mangle* showed the opposite trend and was greatest in the least urban sites. Moreover, the overall tree species diversity increased, while mangrove diversity decreased with increasing surrounding population density within 200 m. The significance of this model, however, was due to the relatively high species numbers at MPNMAX and could not be repeated when this site was removed from the test.

The other response variables, mostly structural, were best explained by water nitrogen and phosphorus concentrations (Figure 5). Mean height, the 90th height percentile, the standard deviation of the height, and the maximum dbh all increased with increasing concentration of total Kjeldahl nitrogen in surface waters. The height skewness decreased with increasing surface water phosphorus, and the stem density decreased with increasing surface water ammonium. Both basal area and biomass decreased with higher surface water temperature. Finally, canopy cover increased with increasing surrounding water coverage within 200 m, and canopy density increased with higher daily flood frequency.

The standard deviation of height was significantly higher in the most urban sites when compared to the least urban sites (t-test, difference = 0.6 m, *p* < 0.01), as was the maximum tree diameter (t-test, difference = 5.7 cm, *p* < 0.01). Additionally, the most urban sites held more species than both the urban (t-test, difference = 0.7 species per plot, *p* < 0.01) and least urban sites (t-test, difference = 1.3 species per plot, *p* < 0.001).

### 3.4. Multiple Regression

To test for the influence of multiple variables on forest structure and composition, fifty-two thousand candidate models were constructed from all combinations of one, two, or three variables, one each from the groups of land cover, hydrology, and water chemistry metrics (Table 5). Top ranked models by BIC almost always included all three variables and resulting R^2^ values averaged 0.74. Although the mean importance of land cover variables in explaining variance in forest composition metrics was highest at 54%, it was not significantly higher than water quality (mean = 32%) or flooding (mean = 26%) (ANOVA, *p* > 0.25). There was also no significant difference in the relative importance of land cover (mean = 33%), flooding (mean = 19%), or water quality (mean = 32%) variables in explaining structural metrics (ANOVA, *p* > 0.2). Specific urban variables of population density, road and highway density, and urban cover held an average relative importance of 28%, compared to 40% for water chemistry and 32% for flooding, again with no statistical difference between the three.

## 4. Discussion

Results from three separate statistical methods (i.e., Non-metric multidimensional scaling, simple regression, and multiple regression) suggest an indirect influence of urban land use on mangrove forest composition and structure in Puerto Rico, and that this influence is shared between flooding dynamics and water chemistry. Non-metric multidimensional scaling showed a weak influence of the urban index in the ordination of sites based on both compositional and structural metrics. It also showed a weak separation of individual sites and limited grouping, suggesting sites are relatively homogenous in structure and composition and not distinguished by their locations or hydro-geomorphologies. Still, simple regression models showed that forest composition was mostly explained by urban variables (i.e., population density and the urban index) and demonstrated increasing tree diversity but decreasing mangrove diversity with greater urbanness. In structural metrics, however, surface water nitrogen and phosphorus concentrations were the most common top ranked predictors and their shared influence on mangrove forests was further evident in multiple regression. This suggests that urban lands may be influencing these forests, albeit indirectly through pathways that are relatively weakly linked to the urban index.

Tree size, as indicated by the maximum and mean diameters as well as the 90th height percentile and mean height at each site, were most strongly predicted by surface water nitrogen in both simple (Figure 5) and multiple regression (Table 5). In the case of diameter, this translates to trees with higher maximum stem diameters in the most urban forests, which were 27% larger than in the least urban forests. This may be because the most urban mangroves are permanently or periodically flooded (0.001–2.5 times per day) by urban waters, which likely carry elevated nitrogen and phosphorus concentrations that have been tied to roads and sewage [76–78]. Such nutrient enrichment has been shown to increase girth but decrease height of mangrove seedlings [79], while the height of dwarf mangroves was found to increase with both nitrogen and phosphorus fertilization [80]. Thus, these nutrients likely influence urban mangrove growth depending upon antecedent conditions and their interaction with other water quality and flooding conditions. Although a previous study at these sites found no linear link between the urban index and surface water nitrogen and phosphorus, it did suggest that urban wastewater effluents may be contributing to elevated concentrations of these elements in the most urban mangrove waters, which held three times as much nitrate and nitrite as the least urban waters [44]. Thus, while there is a relatively weak direct relationship between the urban index and mangrove size, scattered point source wastewater effluents in the most urban sites may indirectly contribute to overall larger trees there.

Despite the positive influence of nitrogen on individual tree size, overall forest structure as measured by stem density was found to be negatively related to ammonium in both simple (Figure 5) and multiple regression (Table 5). This concomitant reduction in stem density with increasing tree size has also been observed in other forests of the Caribbean [51, 81–83], and can be expected from a self-thinning dynamic in which larger trees outcompete smaller surrounding trees, thus reducing stem density and increasing average tree size as the forest matures [51]. Yet, this theory would also predict an overall increase in forest basal area and biomass as stem density is reduced, a pattern we could not detect in the present study (Biomass ~ Stem Density: *p* > 0.1, *R*^2^ < 0.05; Biomass ~ Ammonium: *p* > 0.3, *R*^2^ < 0.1). One study in South Florida suggests that this tradeoff between stem density and biomass occurs only in the absence of stress [82]. Thus, the absence of such a pattern in the present study may be indicative of stress that is limiting forest basal area and biomass, despite sufficient nutrient conditions and larger trees. In fact, water temperature was the strongest predictor of both basal area and biomass in simple and multiple regression and physiological studies have shown an inhibitive effect of high temperatures on mangrove productivity, especially above 25 °C [84–86]. Thus, although the largest trees are larger in urban nitrogen enriched forests, it may be that while overall stem density is driven down by this dynamic, basal area and biomass are driven down by elevated water temperatures (> 30 °C). Perhaps as a result of these confounding factors, there are no differences in forest biomass between the most urban and least urban forests of Puerto Rico.

Compositional metrics were more consistently tied to urbanness than were structural metrics. Overall tree diversity was positively correlated with population density (Figure 5), which agrees with terrestrial urban forests[8]. In the present study, this seems to be driven by the presence of non-mangrove, and in some cases non-native species, found only in the most urban sites, including *Andira inermis*, *Mammea americana*, *Paullinia pinnata*, *Pavonia fruticosa and Tabebuia heterophylla*. The presence of these species within the most urban mangrove forests may be a result of a combination of their increased use in surrounding municipal and residential landscaping [87] as well as lower salinity and inundation due to restricted tidal influence in highly engineered urban landscapes [44]. Little is known on the effect of urban land use on mangrove diversity, but other studies have pointed out the tendency of *L. racemosa* to form monoculture stands in post-disturbance shorelines [15, 88]. This agrees with our results, in which the percent stand biomass composition of *L. racemosa* increased with population density (Figure 5), resulting in roughly 40% more biomass represented by this species at the most urban sites compared to the least urban sites. Contrastingly, the contribution to mangrove biomass by *A. germinans* was negatively related to the urban index and that by *R. mangle* was significantly lower in the least urban sites compared to the most urban sites. For both *L. racemosa* and *R. mangle*, this may be tied to salinity, as both also showed a strong trend with porewater salinity, suggesting that changes in urban hydrology may be driving the observed compositional metrics.

Previous studies have largely hypothesized that observed patterns in urban mangrove species composition are primarily due to changes in hydrology from the urban environment [31, 33]. Indeed tidal influence and connectivity are critical to mangrove zonation and competition with non-halophytes [89, 90]. Thus, it was hypothesized in the present study that changes in the mangrove community along the urban gradient could be tied to changes in tidal connectivity and hydrology. A previous study at these sites did find evidence of reduced tidal amplitudes along the urban gradient as well as immediately upstream of canal restrictions or dredging projects [44]. The present study, however, found mixed evidence in support of this hypothesis. While mangrove composition did change along the urban gradient and a connection to porewater salinity was also observed, these changes were not consistently tied to changes in hydrology or to infrastructure projects. For instance, there was no significant difference in the number of species per plot or the percent stand biomass composition of *A. germinans* or *L. racemosa* between the dredged portion of the Caño Martín Peña at MPDMAX and the un-dredged portion roughly 500 m away at MPNMIN, which exhibits a near 50% (20 cm) reduction in the diurnal tide range. The same is true for SUAMAX and SUAMIN, the two sites on either side of the Suárez canal restriction at the Baldorioty expressway, which also exhibit a 14 cm difference in diurnal tide ranges between the two. Thus, changes in hydrology as a result of infrastructural projects alone cannot account for the observed change in compositional metrics along the urban gradient. Furthermore, while inundation parameters and soil porewater salinity were important in describing variability in forest composition under multiple regression, this importance was shared with and often shadowed by that attributed to surrounding urban metrics. As a result, it cannot be definitively concluded that hydrology or infrastructure projects are the most important drivers of urban mangrove floral community composition. Instead, there may be an alternative selection pressure in urban environments (e.g., residential and municipal landscaping) that drives the observed patterns, which may have implications for the suitability of urban mangroves to provide habitat to a wide variety of taxa that often seek specific floral species [91].

In multiple regression, there was no statistical difference in the average relative importance between variables of urban, water chemistry, and flooding in explaining the observed variation in forest composition and structure. Furthermore, the combination of all three variables in the models explained 70% of the variation of the data, on average, leaving 30% to be explained by other non-measured factors. Thus, while water quality and flooding are important in the structure and composition of mangrove forests, as has been demonstrated in multiple previous studies, land cover and urban intensity may also play a role and should be considered in urban mangrove studies.

## 5. Conclusions

Urbanization presents a conservation and management challenge along tropical coastlines and especially in Caribbean mangrove systems [4, 92, 93]. Not only are mangrove forests lost more quickly around some of the largest cities in the world, but there is growing evidence of systemic changes to ecological function and ecosystem service provisioning in the remaining urban stands [14, 43]. Using quantified metrics of urbanness, the present study corroborated these observations of novel urban mangroves by showing the most urban forests are characterized by higher maximum stem diameters, more species, and fewer true-mangrove species. The inclusion of flooding and water chemistry metrics further showed that nitrogen may be an important driver of forest structure in eutrophic urban waters, that extreme high water temperatures (unrelated to urbanness) may be inhibiting stand basal area and biomass, that the relative abundance of certain species is linked to porewater salinity, which may be driven by changes in tidal connectivity along the urban gradient but that changes in hydrology due to urban infrastructure projects cannot fully account for the observed patterns in floral composition. Thus, the compounding environmental complexities of both the mangrove biome and urban landscapes create a novel suite of ecosystem drivers, the understanding of which demands well-designed and focused experiments and field studies. Furthermore, the importance of this understanding is elevated by a greater demand for valuable mangrove ecosystem services in densely populated cities. Services such as shoreline protection and fisheries support, for example, may not be well provisioned in thin urban forests dominated by *Laguncularia spp.* and lacking the more valuable (for these services) *Rhizophora spp.* and *Avicennia spp.* [94–96]. Thus, managing urban mangroves towards more sustainable social-ecological systems requires not only the conservation of remaining stands, but also a greater understanding of the influence the urban landscape has on mangrove ecology. Future work in these forests should utilize objective and quantified urban metrics for comparisons across sites, and may assess faunal communities, ecophysiology, and biogeochemistry, among others, for a more complete understanding of urban mangrove ecology and more informed management of mangroves in the Anthropocene.

## Figures and Tables

**Table 1.** Site abbreviations used throughout the study and their corresponding locations as well select urban variables from a 1000 m sampling radius, as adapted from Branoff [45]. The urban index is a unitless metric of urbanness that incorporates several variables, with 100 being the most urban and 0 the least urban site relative to the others. Urban and mangrove cover are km^2^ km^−2^, street density is km km^−2^, population density is people km^−2^, and the ratio of urban to mangrove cover (Urban:Mang) is unitless.

Watershed	Site	Lon.	Lat.	Urban Index	Urban Cover	Street Dens.	Pop. Dens.	Mangrove Cover	Urban: Mangrove
**San Juan Bay Estuary** **Río Bayamón/ Río Hondo** **to The Río Puerto Nuevo/ Río Piedras**	BAHMIN	−66.1302	18.44985	29	0.09	1.1	301.3	0.04	2.7
	BAHMAX	−66.0827	18.44436	63	0.45	6.4	2330.7	0.10	4.5
	MPDMIN	−66.0788	18.43787	60	0.44	6.1	1338.2	0.20	2.1
	MPDMAX	−66.0636	18.43329	73	0.59	11.3	3450.0	0.16	3.6
	MPNMIN	−66.0591	18.43373	82	0.65	15.3	4317.9	0.11	6.1
	MPNMAX	−66.0498	18.4307	98	0.75	19.4	7253.6	0.05	13.7
	SANMAX	−66.0342	18.44253	46	0.30	5.3	2568.7	0.13	2.3
	SANMIN	−66.0125	18.41686	35	0.21	4.3	1418.0	0.09	2.3
	SUAMIN	−65.9957	18.42838	56	0.54	3.6	1460.4	0.10	5.4
	SUAMAX	−65.9881	18.42849	64	0.60	6.8	2426.6	0.10	6.1
	TORMIN	−65.9829	18.44735	14	0.10	1.3	376.2	0.32	0.3
	TORMAX	−65.9782	18.43906	19	0.16	3.4	606.8	0.33	0.5
	PINMAX	−65.9570	18.44315	5	0.06	1.8	115.2	0.55	0.1
	PINMIN	−65.9572	18.43104	1	0.00	0.3	0.0	0.50	0.0
**Levittown Río de la Plata**	LEVMIN	−66.2068	18.46477	9	0.02	0.7	29.1	0.42	0.0
	LEVMID	−66.1961	18.45779	24	0.18	4.1	1942.2	0.41	0.4
	LEVMAX	−66.1906	18.45468	48	0.40	9.3	2597.0	0.23	1.8
**Ponce - Río Inabón to the Río Loco**	PONMID	−66.6716	17.97408	31	0.13	1.4	321.2	0.12	1.0
	PONMAX	−66.6095	17.97253	42	0.37	3.1	598.8	0.15	2.4
	PONMIN	−66.5833	17.96285	21	0.00	0.3	0.2	0.19	0.0

**Table 2.** variables used to describe forest structure and composition.

Variable		Units	Description	Source
**Ground**	dbh	cm	The diameter at breast height of each woody stem within the plot	Present study
	Stem density	stems/ha	The number of stems over 1 cm in dbh within the plot over the area of the plot	Present study
	Basal area	m^2^/ha	The sum of cross-sectional areas: $\pi {\left(\frac{dbh}{2}\right)}^{2}$ of all trees in the plot over the area of the plot	Present study
	Biomass	Mg/ha	The sum of biomass estimates from allometric equations for all trees in the plot over the area of the plot	Present study
	Percent stand biomass composition of spp.	%	The total biomass of a species at a plot over the total biomass of all species at that plot	Present study
	Diversity	n	The total number of all species encountered in a plot	Present study
	Mangrove Diversity	n	The total number of all true mangrove species encountered in a plot	Present study
	Soil Porewater Salinity	ppt	Porewater salinity measurements at soil depths of 0, 10, and 20 cm	Present study
**LiDAR**	Mean height	m	Mean height of all returns above 1.4 m	NASA G-LiHT ^a^
	Height SD	m	Standard deviation of all return heights above 1.4 m	NASA G-LiHT ^a^
	Height percentiles	m	Heights at which 10%, 20%…90%, 100% of returns are lower	NASA G-LiHT ^a^
	Height skewness	m	Skewness of all return heights above 1.4 m, a measure of vertical height complexity	NASA G-LiHT ^a^
	Canopy cover	%	Number of first returns above 2 m over the total number of first returns	NASA G-LiHT ^a^
	Canopy density	%	Number of all returns above 2 m over the total number of all returns	NASA G-LiHT ^a^

a Cook et al. 2013 [56].

**Table 3.** Variables used to explain the variation in forest structure and composition metrics described in Table 2.

	Variable	Units	Description	Source
**Urbanness**	Urban cover	km^2^/km^2^	Aerial coverage of all urban and developed open space classes	LULC raster ^a^
	Green and blue cover	km^2^/km^2^	Aerial coverage of all open water and vegetation classes	
	Mangrove cover	km^2^/km^2^	Aerial coverage of all estuarine forested and estuarine scrub–shrub wetland classes	
	Road density	km/km^2^	Length of roads over area of sampling circle	Road network vector ^b^
	Population density	people/km^2^	Number of people within the sampling circle over area of sampling circle	Census tract total population ^c^
	Urban index	-	Normalized mean of the above urbanization variables such that 0 is the least urban and 100 is the most urban value	Above urbanness variables ^d^
**Hydrology**	Average depth	m	Mean depth of water at the site from 2012 to 2017 based on water level models	Water level models ^d^
	Proportion of time flooded	%	Proportion of time with positive depth at the site from 2012 to 2017 based on water level models	
	Mean daily flood frequency	floods/day	Mean number of times per day a positive depth was estimated at the site from 2012 to 2017 based on water level models	
	Mean flood length	day	Mean length of positive depth estimated at the site from 2012 to 2017 based on water level models	
**Water chemistry**	Dissolved oxygen	mg/L	Mean concentration of dissolved oxygen in surface waters measured monthly between 2012 and 2017	San Juan Bay Estuary Program ^e^
	pH	-	Mean pH in surface waters measured monthly between 2012 and 2017	
	Salinity	PSS	Mean salinity in surface waters measured monthly between 2012 and 2017	
	Temperature	°C	Mean temperature in surface waters measured monthly between 2012 and 2017	
	Ammonium	mg/L	Mean concentration of ammonium in surface waters measured bi-annually between 2012 and 2017	
	Total Kjeldahl Nitrogen	mg/L	Mean concentration of total Kjeldahl nitrogen in surface waters measured bi-annually between 2012 and 2017	
	Nitrate and nitrite	mg/L	Mean combined concentration of nitrate and nitrite in surface waters measured bi-annually between 2012 and 2017	
	Total phosphorus	mg/L	Mean concentration of total phosphorus in surface waters measured bi-annually between 2012 and 2017	

a Office for Coastal Management, 2017 [67];

b U.S. Census Bureau, 2015 [68];

c U.S. Census Bureau, 2010 [69];

d Branoff, 2020 [44];

e estuario.org.

**Table 4.** Average and standard error of stem, basal area, and biomass per hectare of all species within the three watersheds included in the study, ordered by average stem density across sites. One individual each of seven additional non-identified species were found in one plot of one site in the San Juan Bay Estuary. Halophytic plant types and salinity tolerances (ppt) provided as superscripts and obtained from Santos et al. [50], when available.

	San Juan Bay Estuary			Levittown			Ponce			All		
Species	Stems / ha	Basal Area m^2^ / ha	Biomass Mg / ha	Stems / ha	Basal Area m^2^ / ha	Biomass Mg / ha	Stems / ha	Basal Area m^2^ / ha	Biomass Mg / ha	Stems / ha	Basal Area m^2^ / ha	Biomass Mg / ha
**All**	5758 ± 282.5	38.6 ± 1.6	207.9 ± 9.8	7724.3 ± 1242.8	42.6 ± 4.7	245.6 ± 33.5	5356.4 ± 632.1	27.7 ± 2.2	156 ± 14.2	5997.1 ± 291.2	37.6 ± 1.4	206 ± 8.9
***Laguncularia racemosa***^†22^	2979.4 ± 275.8	28.1 ± 1.8	144.4 ± 10.1	6695.5 ± 1350.3	33.9 ± 4.4	186.4 ± 29.2	2569.6 ± 719.4	16.2 ± 3.8	86.1 ± 22.2	3556.9 ± 329.3	27.6 ± 1.6	144.1 ± 9.2
***Rhizophora mangle***^†SW^	2219.9 ± 189.3	9.2 ± 1	54.7 ± 7.8	463 ± 94.5	11.3 ± 3.8	101.5 ± 39.3	4165.3 ± 641.6	19.7 ± 1.7	115.4 ± 11.2	2366.3 ± 187.4	10.9 ± 0.9	66.9 ± 7.1
***Avicennia germinans***^†67^	2062.6 ± 383.7	11.3 ± 1.5	59.7 ± 8.2	--	--	--	827.6 ± 355.3	3.5 ± 2	22.4 ± 16.1	1876.2 ± 334.8	10.1 ± 1.3	54.1 ± 7.5
***Thespesia populnea***^†SW^	2132.7 ± 524.7	7.3 ± 2.3	35 ± 11.7	3910.7 ± 687.2	19.5 ± 4	94.8 ± 22.4	1082.3 ± 191	0.2 ± 0	0.4 ± 0.1	2412.3 ± 432.8	9.3 ± 2.1	44.5 ± 10.6
***Calophyllum sp.***	1122.8 ± 323.8	1.9 ± 0.8	6.7 ± 3	286.5 ± 120.5	0.1 ± 0.0	0.2 ± 0.0	--	--	--	899.8 ± 256	1.4 ± 0.6	5 ± 2.3
***Dalbergia ecastaphyllum***^†22^	615.4 ± 300.3	0.5 ± 0.3	1.4 ± 0.8	418.4 ± 110.1	0.6 ± 0.2	2.1 ± 0.6	--	--	--	509.3 ± 146.3	0.6 ± 0.2	1.8 ± 0.5
***Bucida buceras***	382 ± 76.8	0.5 ± 0.1	1.7 ± 0.5	--	--	--	--	--	--	382 ± 76.8	0.5 ± 0.1	1.7 ± 0.5
***unknown fabaceae***	848.8 ± 404.9	6 ± 3.1	30.4 ± 16	--	--	--	--	--	--	848.8 ± 404.9	6 ± 3.1	30.4 ± 16
***Jacquinia arborea***	--	--	--	--	--	--	483.8 ± 84.5	0.2 ± 0.1	0.5 ± 0.3	483.8 ± 84.5	0.2 ± 0.1	0.5 ± 0.3
***Roystonea borinquena***	291 ± 86.6	11.8 ± 6.4	88.6 ± 51.1	127.3 ± --	2.9 ± --	16.3 ± --	--	--	--	270.6 ± 77.7	10.7 ± 5.7	79.6 ± 45.1
***Schefflera morototoni***	466.9 ± 278.3	1.1 ± 0.8	4.1 ± 2.8	127.3 ± --	0.1 ± --	0.2 ± --	--	--	--	382 ± 214.3	0.9 ± 0.6	3.1 ± 2.2
***Terminalia catappa***^†^	350.1 ± 131.2	7.9 ± 3.5	52.5 ± 23.5	127.3 ± --	2.7 ± --	15.1 ± --	--	--	--	305.6 ± 111	6.9 ± 2.9	45 ± 19.7
***Ardisia elliptica***^†^	466.9 ± 212.2	0.1 ± 0.1	0.4 ± 0.3	--	--	--	--	--	--	466.9 ± 212.2	0.1 ± 0.1	0.4 ± 0.3
***Cocos nucifera***^δ6^	169.8 ± 42.4	7.6 ± 1.7	54.7 ± 13.8	127.3 ± 0.0	7.6 ± 0.9	53.6 ± 7.3	--	--	--	159.2 ± 31.8	7.6 ± 1.3	54.4 ± 10.2
***Mammea americana***	159.2 ± 31.8	0.1 ± 0.1	0.3 ± 0.2	--	--	--	--	--	--	159.2 ± 31.8	0.1 ± 0.1	0.3 ± 0.2
***Andira inermis***	254.6 ± 127.3	0.1 ± 0.0	0.3 ± 0.2	--	--	--	--	--	--	254.6 ± 127.3	0.1 ± 0	0.3 ± 0.2
***Coccoloba uvifera***^δ32^	509.3 ± --	0.3 ± --	0.8 ± --	--	--	--	--	--	--	509.3 ± --	0.3 ± --	0.8 ± --
***Tabebuia heterophylla***	509.3 ± --	0.2 ± --	0.5 ± --	--	--	--	--	--	--	509.3 ± --	0.2 ± --	0.5 ± --
***Annona glabra***	--	--	--	382 ± --	5.4 ± --	27.3 ± --	--	--	--	382 ± --	5.4 ± --	27.3 ± --
***Conocarpus erectus***^†SW^	254.6 ± --	11 ± --	76 ± --	--	--	--	127.3 ± --	3.8 ± --	23.2 ± --	191 ± 63.7	7.4 ± 3.6	49.6 ± 26.4
***Hippomane mancinella***^δ^	382 ± --	0.1 ± --	0.2 ± --	--	--	--	--	--	--	382 ± --	0.1 ± --	0.2 ± --
***Pavonia fruticosa***	382 ± --	0.1 ± --	0.2 ± --	--	--	--	--	--	--	382 ± --	0.1 ± --	0.2 ± --
***Paullinia pinnata***	127.3 ± 0	0.9 ± 0.9	4.7 ± 4.5	--	--	--	--	--	--	127.3 ± 0	0.9 ± 0.9	4.7 ± 4.5

Plant type:

† Hydrohalophyte,

δ Psammaphile.

Salinity tolerance (ppt): SW seawater salinity [50].

**Table 5:** Top models for forest structure and composition metrics as determined by the lowest BIC for models containing all possible combinations of surrounding land cover, hydrology, and water quality. Relative importance is the percentage contribution of each variable to the R^2^ value.

					Relative Importance		
Response	Model: Response ~ Land Cover + Flooding + Water Chemistry	BIC	R^2^	p	Land Cover	Water Chem	Flooding
**Stem Density (ha^−1^)**	~ 7500 * ln(Mangrove Cover Within 500 m) - 3549 * Ammonium	225	0.61	0.001	30	70**	--
**Basal Area (m^2^ ha^−1^)**	~ −0.003 * Population Density Within 1 km - 8.8 * Temperature + 5e-04 * Flood Length	78	0.76	0.0004	24**	58***	18*
**Above ground Biomass (Mg ha^−1^)**	~ 107 * log(Vegetation Cover Within 1 km) - 66 * Temperature + 0.001 * Flood Length	116	0.82	0.02	19**	68***	13
**Max DBH (cm)**	~ 2 * log(Population Density Within 1 km) + 2.4 * log(Total Kjeldahl Nitrogen) - 0.004 * Flood Days per Year	62	0.84	0.0004	18**	54***	28*
**Mean DBH (cm)**	~ 0.23 * log(Population Density Within 100 m) + 5.32 * Nitrate & Nitrite - 0.23 * log(Dry Length)	40	0.62	0.04	30*	38**	32*
**90^th^ Height Percentile (m)**	~ 14.4 * Mangrove Cover Within 500 m - 0.08 * Dissolved Oxygen - 0.002 * Flood Days per Year	39	0.84	0.0002	48***	28**	24**
**Height SD (m)**	~ −1.5 * log(Open Water Within 500 m) + 0.13 * Total Kjeldahl Nitrogen - 3e-04 * Flood Days per Year	3	0.77	0.0002	33*	48***	19
**Canopy Cover (%)**	~ 2.9 * log(Open Water Within 1 km) + 5.44 * Nitrate & Nitrite - 0.001 * Flood Length	39	0.82	0.0005	53**	20**	28*
**Canopy Density (%)**	~ 1612* Urban Cover Within 50 m – 4.61*log(Soil Salinity 0 cm) + 8.84*Daily Flood Frequency	66	0.61	0.01	24**	20*	56**
**Percent stand biomass composition of *A. germinans***	~ −67.13 * log(Urban Cover Within 500 m) + 17.77 * Temperature - 1.5 * log(Flood Length)	97	0.85	0.0001	28**	66***	6*
**Percent stand biomass composition of *R. mangle***	−11.5 * log(Urban Index Within 200 m) + 1 * Soil Salinity at 20 cm	125	0.55	0.005	52*	--	48*
***L. racemosa***	~ −583 * log(Open Water Within 200 m) - 1.52 * Salinity - 145.5 * Max Depth	103	0.86	0.0002	15***	16***	68***
**Percent stand biomass composition of non-mangrove**	~ 18150 * Open Water Within 50 m - 25.3 * log(Nitrate & Nitrite) + 15.04 * log(Mean Depth+1)	82	0.76	0.001	88***	8	4
**Tree Diversity**	~ 1136 * log(Open Water Within 50 m) + 0.23 * Temperature - 0.34 * Daily Flood Frequency	7	0.74	0.002	78***	7*	14**
**Mangrove Diversity**	~ −1.31 * log(Urban Cover Within 500 m) + 3.93 * log(Phosphorus) + 0.06 * log(Dry Length)	−1	0.72	0.002	54*	29**	17*

Asterisks are p values of * < 0.05, **<0.01 & *** <0.001.
